# A four gene signature of chromosome instability (CIN4) predicts for benefit from taxanes in the NCIC-CTG MA21 clinical trial

**DOI:** 10.18632/oncotarget.8542

**Published:** 2016-04-01

**Authors:** Melanie Spears, Nicola Lyttle, Alister D'Costa, Bingshu E. Chen, Cindy Q. Yao, Paul C. Boutros, Margot Burnell, Mark N. Levine, Patti O'Brien, Lois Shepherd, John M.S. Bartlett

**Affiliations:** ^1^ Transformative Pathology, Ontario Institute for Cancer Research, MaRS Centre, Toronto, ON, Canada; ^2^ Department of Laboratory Medicine and Pathobiology, University of Toronto, Toronto, ON, Canada; ^3^ Informatics and Bio-Computing, Ontario Institute for Cancer Research, MaRS Centre, Toronto, ON, Canada; ^4^ NCIC Clinical Trials Group (NCIC CTG) and Queen's University, Kingston, ON, Canada; ^5^ Department of Medical Biophysics, University of Toronto, Toronto, ON Canada; ^6^ Department of Pharmacology & Toxicology, University of Toronto, Toronto, ON Canada; ^7^ Saint John Regional Hospital, Saint John, MB, Canada; ^8^ Ontario Clinical Oncology Group, McMaster University, Hamilton, ON, Canada; ^9^ Edinburgh Cancer Research UK Centre, MRC IGMM, University of Edinburgh, Edinburgh, UK

**Keywords:** breast cancer, chromosome instability, predictive biomarker, anthracycline, taxane

## Abstract

Recent evidence demonstrated CIN4 as a predictive marker of anthracycline benefit in early breast cancer. An analysis of the NCIC CTG MA.21 clinical trial was performed to test the role of existing CIN gene expression signatures as prognostic and predictive markers in the context of taxane based chemotherapy.

RNA was extracted from patients in cyclophosphamide, epirubicin and fluorouracil (CEF) and epirubicin, cyclophosphamide and paclitaxel (EC/T) arms of the NCIC CTG MA.21 trial and analysed using NanoString technology.

After multivariate analysis both high CIN25 and CIN70 score was significantly associated with an increased in RFS (HR 1.76, 95%CI 1.07-2.86, p=0.0018 and HR 1.59, 95%CI 1.12-2.25, p=0.0096 respectively). Patients whose tumours had low CIN4 gene expression scores were associated with an increase in RFS (HR: 0.64, 95% CI 0.39-1.03, p=0.06) when treated with EC/T compared to patients treated with CEF.

In conclusion we have demonstrated CIN25 and CIN70 as prognostic markers in breast cancer and that CIN4 is a potential predictive maker of benefit from taxane treatment.

## INTRODUCTION

Taxanes and anthracyclines are widely used for the treatment of breast cancer yet highly toxic to patients [1–4]. At present there is no biological marker or assay available to identify which subset of patients will benefit from chemotherapy although numerous molecules have been investigated with limited success [4–12]. Research from our group [8] linked the predictive effect of CEP17 *in vivo* to chromosome instability (CIN), which itself is predictive of anthracycline benefit in the BR9601 trial [8]. We recently derived a four gene signature that was predictive of anthracycline benefit and demonstrated that patients with low tumour CIN4 scores benefited from anthracycline treatment significantly more than those with high CIN4 scores (HR 0.37, 95% CI 0.20-0.56, p=0.001) [13]. Given that most patients now receive taxane based chemotherapy we sought to extend the validation of this marker into trials including taxanes.

The NCIC Clinical Trials Group (CTG) MA.21 trial compared dose-intense cyclophosphamide, epirubicin and fluorouracil (CEF) to dose-dense, dose-intense epirubicin, cyclophosphamide with added paclitaxel (EC/T) and to a standard at the time of doxorubicin, cyclophosphamide and paclitaxel (AC/T). Analyses comparing the two epirubicin arms of the trial (CEF vs EC/T) did not demonstrate benefit from the addition of taxane (EC/T vs CEF) in terms of relapse free survival (RFS) (HR 0.85, 95% CI 0.64-1.22, p=0.46) [14].

Our present study, we evaluated the predictive effect of the CIN signatures in patients in the NCIC CTG MA.21 trial and hypothesized that CIN signatures are associated with clinical outcome and resistance to taxane therapy.

## RESULTS

### Characteristics

**Figure 1:**
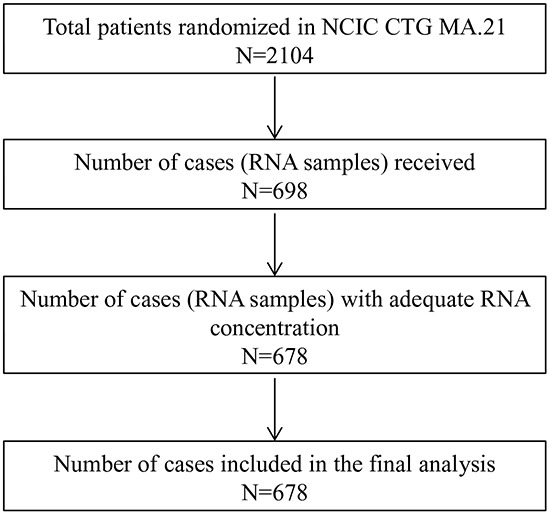
REMARK diagram for the evaluation of chromosome instability (CIN) gene expression signatures in the NCIC CTG MA.21 trial

Table 1 shows the baseline characteristics of the MA.21 CIN study population, whereas Figure 1 illustrates the total number of cases available for analysis; 342 RNA samples from CEF arm and 336 RNA samples from the ECT arm were successfully analysed on the NanoString platform. High CIN4, CIN25 and CIN70 scores were defined as above the median and were previously described [15–13].

**Table 1:** Baseline patient and tumour characteristics from the MA.21 study

Baseline characteristics	CEF 342 (100%)	ECT 336 (100%)
Age		
≤39	56 (16.4%)	59 (17.6%)
40-49	159 (46.5%)	146 (43.4%)
50-59	126 (36.8%)	128 (38.1%)
60-69	1 (0.3%)	3 (0.9%)
Median		
Menopausal status		
Post-menopausal	102 (29.8%)	101 (30.1%)
Pre-menopausal	240 (70.2%)	235 (69.9%)
#of positive axillary nodes		
0	109 (31.9%)	102 (30.4%)
1-3	136 (39.8%)	146 (43.5%)
4-10	79 (23.0%)	72 (21.4%)
>10	18 (5.3%)	16 (4.7%)
Tumour stage		
T1	108 (31.6%)	114 (34.2%)
T2	200 (58.5%)	177 (53.2%)
T3	30 (8.8%)	37 (11.1%)
T4	4 (1.1%)	5 (1.5%)
Missing		3
ER Status		
Positive	195 (57.0%)	196 (58.3%)
Negative	147 (43.0%)	140 (41.7%)
CIN4		
High	161 (47.1%)	164 (48.9%)
Low	181 (52.9%)	173 (51.1%)
CIN25		
High	183 (53.5%)	186 (55.2%)
Low	159 (46.5%)	151 (44.8%)
CIN70		
High	181 (52.9%)	186 (55.2%)
Low	161 (47.1%)	151 (44.8%)

### Correlation of CIN signatures and clinicopathological parameters with clinical outcomes

In univariate analysis using continuous clinico-pathological biomarkers, high CIN4 was associated with ER positivity (p<0.0001) and grade (p<0.001), whereas CIN25 and CIN70 were associated with younger age (p=0.01 and p=0.001, respectively), increase in the number of positive axillary nodes (p<0.0001) and ER negativity (p<0.0001) (Table 2).

**Table 2:** Correlations between CIN scores and conventional pathological markers

	CIN4	CIN25	CIN70
Age	NS	**-0.128** **p=0.01**	**-0.131** **p=0.001**
Positive axillary node	NS	**-0.246** **p<0.001**	**-0.226** **p<0.001**
Tumour size	NS	NS	NS
Grade	**-0.195** **p<0.001**	**0.528** **p<0.001**	**0.500** **p<0.001**
ER	**0.092** **p=0.017**	**-0.430** **p<0.001**	**-0.413** **p<0.001**
HER2	NS	NS	NS

### CIN signatures as a prognostic marker for RFS

**Figure 2:**
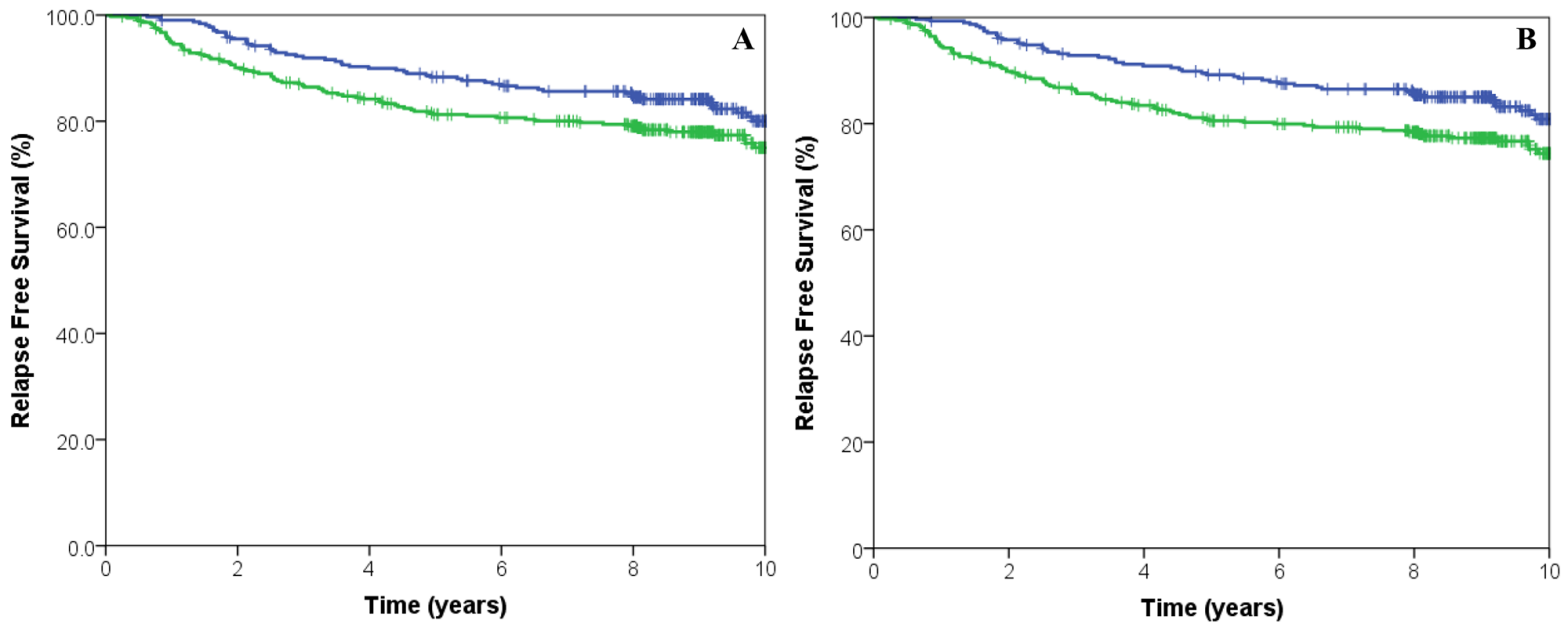
Kaplan-Meier survival curves for low CIN70 score (green line) and high CIN70 score (blue line) for relapse free survival **A.** Kaplan-Meier survival curves for low CIN25 score (green line) and high CIN25 score (blue line) for relapse free survival **B.**

The prognostic impact of the CIN signatures were tested on the entire cohort, irrespective of allocated adjuvant chemotherapy. No statistically significant association was observed between CIN4 or CIN70 scores and RFS (HR: 1.11, 95% CI 0.79-1.55, p=0.548 and HR: 0.73, 95% CI 0.52-1.02, p=0.07, (Figure 2A) respectively). In contrast, tumours with high CIN25 scores were associated with increased RFS (HR: 1.52, 95%CI 2.13-10.64, p=0.02) (Figure 2B). After multivariate analysis and adjustment for nodal status, grade, size, age, HER2 and ER status, both high CIN25 and CIN70 scores were significantly associated with an increased RFS (HR 1.76, 95%CI 1.07-2.86, p=0.0018 and HR 1.59, 95%CI 1.12-2.25, p=0.0096 respectively).

### CIN signatures as predictive markers of taxane benefit

Subsequent analysis focused on the differential impact of multigene signatures on RFS between patients receiving anthracycline plus taxane (EC/T) therapy and those given anthracycline treatment (CEF) alone. Univariate analysis demonstrated no significant difference in benefit from ECT versus CEF treatment between patients whose tumours had high (HR 0.79, 95%CI 0.51-1.23, p=0.30) or low CIN70 expression (HR 1.30, 95% CI 0.76-2.21, p=0.34) (Table 3). Similarly there was no differential benefit from ECT treatment between patients whose tumours had high (HR 0.78, 95%CI 0.51-1.20, p=0.26) or low CIN25 expression (HR 1.35, 95% CI 0.78-2.34, p=0.28).

**Table 3:** Hazard ratios for relapse free survival comparing cyclophosphamide, epirubicin and fluorouracil (CEF) with cyclophosphamide, epirubicin and paclitaxel (EC/T) by biomarker status

	Relapse Free Survival					
	Low Biomarker		High Biomarker		Treatment*Marker	
	HR	95% CI	HR	95% CI	HR	Test for Interaction *P*
CIN70	1.30	0.76-2.21	0.79	0.51-1.23	1.43	0.355
CIN25	1.35	0.78-2.34	0.78	0.51-1.20	1.57	0.263
CIN4	0.64	0.39-1.03	1.53	0.91-2.57	0.49	0.066

**Figure 3:**
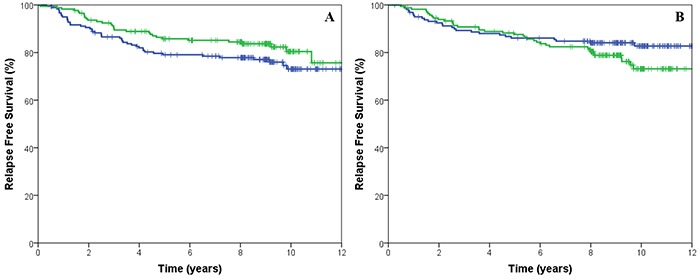
Kaplan-Meier survival curves for cyclophosphamide, epirubicin and fluorouracil (CEF, blue) treated with cyclophosphamide, epirubicin and paclitaxel (EC/T, green line) for relapse free survival stratified by low CIN4 expression **A.** and high CIN4 expression **B.**

The hazard ratio for treatment by marker interaction of CIN25 and CIN70 before correction for clinical variables was 0.58 (95% CI 0.29-1.17, p=0.128) and 0.61 (95% CI 0.31-1.122, p=0.166), respectively. After correction for size, nodal status, ER status, HER2, grade, CIN4 and treatment, the hazard ratio was 0.64 (95% CI 0.29-1.40, p=0.263) and 0.70 (95% CI 0.33-1.50, p=0.355) for CIN25 and CIN70, respectively. However, a trend was noted in patients whose tumours had low CIN4 gene expression scores with an increase in RFS (HR: 0.64, 95% CI 0.39-1.03, p=0.06) when treated with EC/T compared to patients treated with CEF (Figure 3). There was no statistical significant difference in survival in patients that has high CIN4 gene expression scores (HR: 1.54, 95% CI 0.1-2.57, p=0.107). The hazard ratio for treatment by marker interaction of CIN4 before correction for clinical variables was 0.49 (95% CI 0.25-0.97, p=0.04) for RFS. After correction for size, nodal status, ER status, HER2, grade, CIN4 and treatment, the hazard ratio was 0.49 (95% CI 0.23-1.05, p=0.066).

## DISCUSSION

CIN is associated with poor prognosis in patients with solid tumours [16–15]. We have previously shown that CIN is associated with sensitivity to anthracycline treatments [8–13] while others have demonstrated that CIN is associated with taxane resistance [17]. Since modern chemotherapy treatment of breast cancer includes the addition of a taxane, we examined the role of three CIN gene signatures in the NCIC CTG MA.21 breast cancer trial that compares taxane versus no-taxane containing arms. Our study has demonstrated that patients with low expression of the CIN4 gene signature exhibited greater benefit from the EC/T treatment regimen versus the CEF regimen. This is in contradiction to previous work in ovarian cancer suggesting a CIN70 gene expression signature was predictive of resistance to taxanes and sensitivity to carboplatin treatment [17]. However, it is unclear if our current result reflects the addition of a taxane to the treatment regimen or simply extending the duration of the chemotherapy regimen. Both arms included a cumulative dose of 720mg/m^2^ of epirubicin however; the EC/T arm had an additional four cycles of paclitaxel [14]. Therefore, our result could reflect either the addition of paclitaxel or the need for extended chemotherapy. However, there is accumulating evidence to support the former. For example, the UK TACT study investigated whether sequential docetaxel after anthracycline chemotherapy would improve patient outcome compared to standard chemotherapy of a similar duration. This study demonstrated no difference in disease free survival between the FEC-D group and the control group (HR 0.95, 95% CI 0.85-1.08, p=0.44) [18]. Meta-analyses performed by the EBCTCG group demonstrated the incorporation of taxanes into an anthracycline regimen resulted in the reduction of the risk of recurrence and survival at 8 years [19]. This analysis recognised there was variation in trial designs when comparing the effects of additional cycles of just a taxane to a constant background chemotherapy regimen. However, in the taxane trials with different number of cycles the 8-years breast cancer mortality was 21.1% for the taxane groups versus 23.9% for the control groups. In contrast in trials examining the effects of a taxane regimen with the same number of cycles non-taxane chemotherapy demonstrated the 5-year a small but significant reduction in breast cancer mortality [19]. These results would indicate that the addition of a taxane to the chemotherapy regimen improves survival and the CIN4 is predictive of taxane benefit.

In agreement with other studies, we have demonstrated that the CIN25 and CIN70 signature is an independent prognostic biomarker in breast cancer [15–20]. We performed our analysis on the NanoString platform, which has been shown to be a robust and reproducible platform that can easily be incorporated into the pathology laboratory setting [21]. The identification of methods that predict breast cancer clinical outcome is being studied in numerous labs around the world. There are a number of prognostic expression arrays available such as Oncotype DX [22], Mammaprint [23] and Prosigna [21] that predict patient outcome in estrogen receptor (ER) positive patients; however, none of them are tailored towards the ER negative population. The CIN25 and CIN70 gene expression signatures were derived irrespective of ER status and contain genes, such as *TPX2*, *TOP2A*, *MAD2L1*, *CCNB1*, *CDC20* and *PTTG1* that are involved in spindle assembly checkpoint (SAC) as well as DNA damage checkpoint genes [15]. Therefore, the CIN25 and CIN70 signatures are credible candidates for use in the prognostic setting.

Ultimately, although there are a number of prognostic markers available, but there are no predictive signatures associated with specific chemotherapies to level I evidence. We had previously demonstrated the CIN4 signature to be predictive of anthracycline benefit [13], in this study we demonstrate that CIN4 has potential to be predictive for benefit from the addition of a taxane to chemotherapy treatment.

## MATERIALS AND METHODS

### Patients

The MA.21 (ClinicalTrials.gov (NCT00014222)) trial recruited 2104 pre- and post-menopausal women with histologically confirmed node positive or high risk node negative adenocarcinoma of the breast treated with either total or partial mastectomy. Patients were randomised to one of three regimens to receive: 1) 6 cycles of CEF (epirubicin (60mg/m^2^) and fluorouracil (500mg/m^2^) given intravenously on days 1 and 8 and oral cyclophosphamide (75mg/m^2^) on days 1-14) every 28 days, 2) 6 cycles of EC/T (epirubicin (120mg/m^2^) and cyclophosphamide (830mg/m^2^) on day 1 and filgrastim (G-CSF) subcutaneously (SC) on days 2-13 followed by 4 cycles of paclitaxel (175mg/m^2^)) every 21 days, 3) 4 cycles of AC/T (doxorubicin (60mg/m^2^) and cyclophosphamide (600mg/m^2^) on day 1 every 21 days followed by 4 cycles of paclitaxel (175mg/m^2^)) every 21 days. Treatment in all arms continued in the absence of disease progression or unacceptable toxicity; patient characteristics are shown in Table 1. The primary end point for the MA.21 trial was relapse-free survival (RFS) defined as the time from randomization to the time of recurrence of the primary disease. The protocol was approved by central and local ethics committees, and each patient provided written informed consent prior to randomization. For the current analysis, tissue blocks were retrieved for RNA extraction.

### RNA extraction

Total RNA from FFPE tissue samples (2 × 10μM sections) was extracted using the RecoverAll Total Nucleic Acid Isolation kit (Life Technologies) according to the manufacturer's protocol and concentrations were determined using the NanoDrop ND-1000 spectrophotometer (NanoDrop Technologies).

### Gene expression analysis

RNA (200ng) was used with the nCounter system, according to the manufacturer's protocol (NanoString ® Technologies, Seattle, WA, USA). In brief, 5μl of RNA was hybridized at 96°C overnight with the NanoString Codeset. Probes for the analysis were synthesized by NanoString technologies and included probes for the 93 genes of interest and 6 normalising genes (Supplementary Table S1); all 99 genes were assayed simultaneously in multiplexed reactions. After probe hybridizations and NanoString nCounter digital reading, counts for each RNA species analyzed. The nCounter CodeSet contains two types of built-in controls: positive controls (spiked RNA at various concentrations to assess the overall assay performance) and negative controls (probes for background calculation). Raw mRNA abundance count data were pre-processed using the NanoStringNorm R package (v1.1.19) using normalization factors derived from the geometric mean of housekeeping genes, mean of negative controls and geometric mean of positive controls [24]. The CIN4/25/70 scores were developed and analysed as described in [13].

### Statistics

The SAS (SAS Institute Inc., Cary, NC, USA, version 9.2) statistical package was used for statistical analysis. Kaplan-Meier curves of survival were used for estimation of relapse free survival (RFS), whereas log-rank test was used to compare RFS among different groups of patients. The Cox proportional hazards model was used to estimate hazard ratios for relapse. When comparing outcomes between the treatment arms within the groups of patients identified by biomarker expression, p-values were not calculated for sub-groups to avoid multiple testing and bias where one group was much smaller than the other. The Cox model was instead used to identify statistically significant interactions (p<0.05) between biomarkers and outcome on the different treatments (treatment by marker interaction), in models that also included biomarker status (marker effect) and treatment, as covariates. In exploratory analyses a value of p<0.05 was considered statistically significant as the Bonferroni correction was not applied.

## SUPPLEMENTARY MATERIALS TABLE


