# Supplementary material for: A four gene signature of chromosome instability (CIN4) predicts for benefit from taxanes in the NCIC-CTG MA21 clinical trial
Source: Oncotarget. 2016 Apr 1;7(31):49099–106. doi: 10.18632/oncotarget.8542 (PMC5226493; doi:10.18632/oncotarget.8542)
Supplement: Supplementary file 1 [file oncotarget-07-49099-s001.pdf]

SUPPLEMENTARY TABLE

Supplementary Table S1: Experimental and housekeeping gens used in the gene expression study

Experimental genes						Housekeeping genes
ACTL6A	CCT5	CKS2	FOXM1	MELK	RFC4	GUSB
14-3-3	CDC2	CMAS	GPIandMGC13096	MSH6	RICTOR	PUM1
APC	CDC20	CNAP1	H2AFX	MTB	RNASEH2A	SF3A1
ASF1B	CDC25B	CTPS	H2AFZ	MTCH2	RRM1	TBP
ATAD2	CDC25C	DHCR7	HDGF	NDUFAB1	RRM2	TFRC
ATM	CDC3A	DKC1	ILK	NEK2	SFRS2	TMED10
ATR	CDC45L	E2F1	KIAA0286	NUP205	TGIF2	
AURKA	CDC6	ECT2	KIF20A	NXT1	TOP2A	
AURKB	CDCA8	ELAV1	KIF4A	OIP5	TOPK	
BRRN1	CDK2	ERK1	LSM4	P53	TPX2	
bub1	CDKN1A	ERK2	MAD2	PAXILLIN	TRIP13	
Bub3	CEP55	ESPL1	MAD2L1	PCNA	TTK	
BuBR1	CHEK1	EZH2	MCM10	PRC1	UBE2C	
ACTL6A	CHEK2	Fak	MCM2	PTTG1	UNG	
CCNB1	CDC2	FEN1	MCM7	RAD21	ZWINT	
CCNB2	ch-TOG	FLJ10036	MDM2	RAD51AP1		
